# MnO_2_@Reduced Graphene Oxide Nanocomposite-Based Electrochemical Sensor for the Simultaneous Determination of Trace Cd(II), Zn(II) and Cu(II) in Water Samples

**DOI:** 10.3390/membranes11070517

**Published:** 2021-07-09

**Authors:** Siyamthanda Hope Mnyipika, Tshimangadzo Saddam Munonde, Philiswa Nosizo Nomngongo

**Affiliations:** 1 Department of Chemical Sciences, University of Johannesburg, Doornfontein Campus, Doornfontein 2028, South Africa; cia.mnyi@gmail.com; 2 Department of Science and Innovation (DSI)/National Research Foundation (NRF) South African Research Chair (SARChI), Nanotechnology for Water, University of Johannesburg, Doornfontein 2028, South Africa; 3 Department of Science and Innovation (DSI)/Mintek Nanotechnology Innovation Centre, University of Johannesburg, Doornfontein 2028, South Africa

**Keywords:** MnO_2_@RGO@GCE, glassy carbon electrode, trace metals, differential pulse anodic stripping voltammetry, surface water

## Abstract

The rapid detection of trace metals is one of the most important aspect in achieving environmental monitoring and protection. Electrochemical sensors remain a key solution for rapid detection of heavy metals in environmental water matrices. This paper reports the fabrication of an electrochemical sensor obtained by the simultaneous electrodeposition of MnO_2_ nanoparticles and RGO nanosheets on the surface of a glassy carbon electrode. The successful electrodeposition was confirmed by the enhanced current response on the cyclic voltammograms. The XRD, HR-SEM/EDX, TEM, FTIR, and BET characterization confirmed the successful synthesis of MnO_2_ nanoparticles, RGO nanosheets, and MnO_2_@RGO nanocomposite. The electrochemical studies results revealed that MnO_2_@RGO@GCE nanocomposite considerably improved the current response on the detection of Zn(II), Cd(II) and Cu(II) ions in surface water. These remarkable improvements were due to the interaction between MnO_2_ nanomaterials and RGO nanosheets. Moreover, the modified sensor electrode portrayed high sensitivity, reproducibility, and stability on the simultaneous determination of Zn(II), Cd(II), and Cu(II) ions. The detection limits of (S/N = 3) ranged from 0.002–0.015 μg L^−1^ for the simultaneous detection of Zn(II), Cd(II), and Cu(II) ions. The results show that MnO_2_@RGO nanocomposite can be successfully used for the early detection of heavy metals with higher sensitivity in water sample analysis.

## 1. Introduction

The key challenge in environmental pollution remediation is the early detection of the contaminants. Superior remediation outcomes are directly acclimated to the early detection of contaminants. Thus, enabling the application of effective treatment processes that reduces cost amongst other resources [1,2]. Consequently, electrochemical sensors can detect various electroactive compounds by providing specific responses to various analytes by generating Faraday currents at different potentials [3]. Heavy metals such as copper (Cu), cadmium (Cd), and zinc (Zn), amongst others, are electroactive materials that are suitable for electrochemical detection. Evidently, trace amounts below the maximum recommended levels by the world Health Organization (WHO), copper and zinc, amongst others, play an important role in living organisms as they are essential for the metabolism related maintenance [1,4]. However, the excessive levels of these heavy metals beyond the WHO permitted levels can cause serious environmental and human health effects [5]. For instance, water containing Cu beyond the prescribed limit of 1.3 mg L^−1^ may cause lung cancer and liver damage in human beings [6]. Cadmium, on the other hand, is utilized in many processes such as batteries and pigments, thus leaching into the water systems occurs after the disposal of the waste or the final products. However, consuming water that has significant amounts of Cd, may result in the accumulation in the human body, thus causing negative effects such as blood pressure complications and kidney [7,8]. Zinc is an important element with significant health benefits at trace levels, but accumulates when exceeding recommended levels, as it is non-biodegradable. Nausea, diarrhea, depression, lethargy, seizures, and a short-term metal-fume fever are some of the effects from ingesting water contaminated with excess Zn [9]. Therefore, due to the serious hazardous effects of heavy metal ions on human health and the toxicity to the ecosystem, it is important to develop a simple and highly sensitive electrochemical method for the early detection of the studied heavy metals, to improve the quality of the environment and human life.

Significant progress has been made with various analytical techniques such as flame atomic absorption spectrometry (FAAS) [10], electrothermal atomic absorption spectrometry (ETAAS) [11], inductively coupled plasma optical emission spectrometry (ICP-OES) [12], and inductively coupled plasma optical mass spectrometry (ICP-MS) [13], on the determination of various heavy metals in different matrices. However, most of the above-mentioned methods have major disadvantages, such as complexity, expensive, time consuming, bulky, require complicate pretreatment, and inconvenient for onsite and in situ analysis [14]. Compared with the abovementioned methods, electrochemical techniques have attracted great interest on the detection of heavy metal ions due to their remarkable sensitivity, portability, and low cost [1,15,16]. For this reason, there is an ongoing research on the development of rapid and friendly-user techniques for trace heavy metals detection suitable for in situ monitoring assays [17]. Among the different electrochemical techniques, stripping voltametric methods are the most reported for heavy metals detection due to their excellent detection limits, sensitivity, ability to determine multielement, and relatively low-cost instrumentation [18,19].

The performance of an electrochemical technique is strongly influenced by the working electrode material. For several decades, electrochemical stripping methods were associated with the use of working mercury electrodes due to their wide cathodic potential range [20]. However, due to the toxicity of mercury, alternative electrodes such as glassy carbon electrode (GCE), amongst others, have been used for electrochemical determination of heavy metals [21]. The main advantages of GCE electrodes are that they are environmentally friendly, cheap, negligible toxicity, analytical properties are comparable to those of mercury electrodes and can be easily modified [22]. Subsequently, some interfering species and impurities from aqueous samples tend to accumulate on the surface of the bare electrode due to their hydrophobic nature or other affinities toward electrode [23]. Therefore, to minimize the limitations that are encountered on the use of GC electrodes for the detection of different analytes, different nanomaterials or nanocomposites have been used to modify the GC electrodes [24,25,26,27,28,29]. Tuning the composition, structure and morphology of the nanomaterials/nanocomposites improves surface properties of the electrodes, thus increasing their sensing element [30,31].

Thus, the aim of the present study was to utilize a GC electrode modified with MnO_2_@RGO nanocomposite for the sensitive simultaneous determination of Cd, Cu, and Zn in surface water using differential pulse anodic stripping voltammetry (DPASV). The MnO_2_ nanomaterials were selected due to their low toxicity, inexpensive, and high electrochemical activity [32], whereas RGO nanosheets were selected due to their high electrical conductivity, surface area, and mechanical properties [33]. The factors affecting the electrochemical determination of the selected analytes were optimized using a central composite design. Additionally, the effect interfering ions, precisions (repeatability and reproducibility), and the accuracy of the developed electrochemical sensor were also investigated.

## 2. Materials and Methods

### 2.1. Reagents and Materials

All reagents and chemicals were of analytical reagent grade and doubly distilled water was used in all the experiments. Graphite flakes, potassium permanganate (KMnO_4_), sulphuric acid (H_2_SO_4_), phosphoric acid (H_3_PO_4_), hydrochloric acid (HCl), hydrogen peroxide (H_2_O_2_), polyvinyl alcohol (PVA), potassium chloride (KCl), hexanol, and absolute ethanol were purchased from Sigma-Aldrich (St. Louis, MO, USA). Spectrascan standard stock solutions of Cd, Cu, and Zn (1000 mg L^−1^, Teknolab, Norway) were used to prepare the working standard solutions for the quantification of analyte concentrations in model solutions. Acetate buffer (1.0 mol L^−1^, pH 5) was used as a supporting electrolyte.

### 2.2. Instrumentation

Electrochemical measurement such as cyclic voltammetry (CV), differential pulse anodic stripping voltammetry (DPASV), and electrochemical impedance spectroscopy (EIS) were performed using a μ-Autolab TYPE III conventional three-electrode system driven by the NOVA software (Version 1.8). A modified glassy carbon electrode was employed as a working electrode and a platinum wire as the counter electrode and Ag/AgCl/KCl (3 mol L^−1^) reference electrode. Electrochemical measurements were performed at room temperature. All pH measurements were done using H1 9811-5, (HANNA Instruments, Smithfield, RI, USA) pH meter supplied with a combined electrode. The morphology, nano structure and particle size of the material were examined using the transmission electron microscopy (TEM, JEM-2100, JEOL, Tokyo, Japan) and scanning electron microscopy (SEM, TESCAN VEGA 3 XMU, LMH instrument (Czech Republic) coupled with energy dispersive X-ray spectroscopy (EDS) for elemental composition analysis at an accelerating voltage of 20 kV. Infrared spectra were recorded (4000–400 cm^−1^), using a Perkin Elmer spectrum 100 Fourier transform infrared spectrometer (Waltham, MA, USA). The XRD measurements were done on a Rigaku Ultima IV X-ray diffractometer using a Cu-Kα radiation (λ = 0.15406 nm). BET measurements were performed on a Tristar II (Micromeritics, Norcross, GA, USA) after the samples were degassed at 77 °C for 16.5 h. All the BET surface area, pore size, and volume were measured using nitrogen at 77 K.

### 2.3. Preparation of Reduced Graphene Oxide (RGO)

Graphene oxide (GO) was initially prepared using the modified Hummers’ method [34,35]. Briefly, H_2_SO_4_ and H_3_PO_4_ were mixed in a 9:1 volume ratio (360:40 mL) and stirred in an ice bath for 30 min. Graphite flakes (3.06 g) and KMnO_4_ (18.21 g) were slowly added to the mixing solution under stirring. The mixture was transferred to an oil bath and heated to 85 °C for 30 min. Then, 50 mL of deionized water was added to the mixture, which was maintained under stirring at the same conditions for 60 min. The mixture was placed in an ice bath, and 150 mL of deionized water and 20 mL of H_2_O_2_ were slowly added to terminate the reaction. An exothermic reaction occurred, and the solution was then allowed to cool down. The resulting solution was diluted with 1 mol L^−1^ HCl solution and centrifuged at 6000 rpm for 20 min. The supernatant was removed, and the residuals were washed several times with deionized water until a neutral pH was obtained (pH 7.05). Finally, the prepared samples were dried in an oven at 80 °C for 12 h. The obtained GO was thermally exfoliated by annealing in a muffle furnace at 500 °C at a heating rate of 2 °C min^−1^ for 2 h and the furnace was allowed to cool to 35 °C. Thus, GO was successfully converted to RGO [35].

### 2.4. Preparation of Manganese Oxide Nanoparticles (MnO_2_)

The synthesis of MnO_2_ nanoparticles (NP) followed a previously modified method [36]. Typically, 6 mL of 0.5 mol L^−1^ KMnO_4_ solution was added into 100 mL of the initially prepared ethanol: deionized water (50 mL:50 mL; 1:1) mixture solvent at room temperature. The mixture was stirred for 30 min before a 10% H_2_O_2_ (10 mL) and polyvinyl alcohol (PVA) (10 mL) were added, forming a brown precipitate. The supernatant was discarded, and the collected brown precipitate was sonicated with a mixture of ethanol (20 mL) and hexanol (20 mL) to remove the PVA. The precipitate was then washed several times with deionized water and a brown powder obtained was dried at 80 °C in an oven.

### 2.5. Preparation of the MnO_2_@RGO/GCE Modified Electrode

The MnO_2_@RGO/GCE modified electrode was fabricated using an electrodeposition method. Initially, the bare GCE was polished successively to a mirror surface using 1.0, 0.3, and 0.05 µm alumina slurries, and then ultrasonically cleaned in deionized water, anhydrous ethanol, and deionized water for 5 min each to remove any organic and inorganic remnants on the electrode surface, prior being air dried at room temperature (24 °C). The electrochemical deposition was achieved by firstly, dispersing 150 mg RGO and 150 mg MnO_2_ in 2 mL 0.1 mol L^−1^ KCl using ultrasonic bath. Then, the pre-cleaned GCE working electrode, Ag/AgCl reference electrode, and a Pt wire counter electrode were immersed in the mixture, and magnetically stirred at a speed of 100 rpm. The MnO_2_@RGO nanocomposite was then electrodeposited onto the GCE surface using cyclic voltammetry (CV) by scanning in the potential range of −0.2 V to +1.2 V with 6 cycles at a scan rate of 25 mV s^−1^. The obtained MnO_2_@RGO/GCE electrode was washed with deionized water before being air dried at room temperature.

## 3. Results

### 3.1. X-ray Diffraction (XRD)

**Figure 1:**
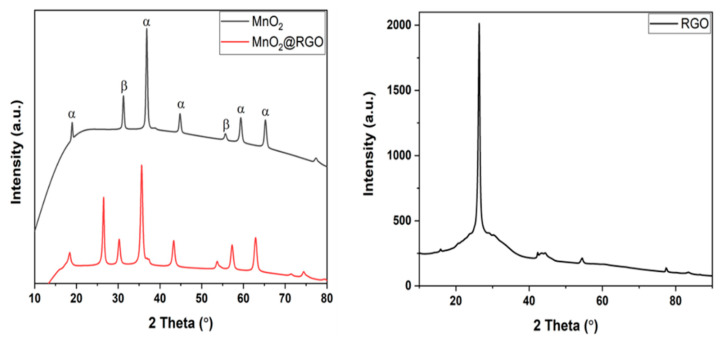
XRD spectra of RGO nanosheets, MnO_2_ nanoparticles and MnO_2_@RGO nanocomposite.

XRD patterns demonstrating the crystal structure of RGO nanosheets, MnO_2_ nanoparticles, and MnO_2_@RGO nanocomposite are displayed in Figure 1. The XRD pattern of MnO_2_ nanoparticles demonstrated the existence of MnO_2_ as a combination of two phases (α and β phases) at 2-theta angles 18.9°, 31.3°, 36.9°, 44.8°, 55.7°, 59.4°, and 65.3°, corresponding to the (200), (110), (211), (301), (600), (220), and (541) planes of typical α (JCPDS No 044-0141) and β (024-0735) phases [37,38,39]. Evidently, the RGO pattern shows an intense sharp peak corresponding to the (002) plane of carbon at 2-theta angle of 26.3° [35]. Expectedly, the RGO nanosheets and MnO_2_ nanoparticles characteristic peaks were observed on the XRD pattern of the MnO_2_@RGO nanocomposite. Interestingly, the MnO_2_@RGO nanocomposite showed major shifts in lower angles of all the peak positions as shown in Figure 1. This suggests the increase in the lattice parameters confirming the change in the crystal structure due to the formation of a nanocomposite [40,41].

### 3.2. High Resolution-Scanning Electron Microscopy (HR-SEM)

**Figure 2:**
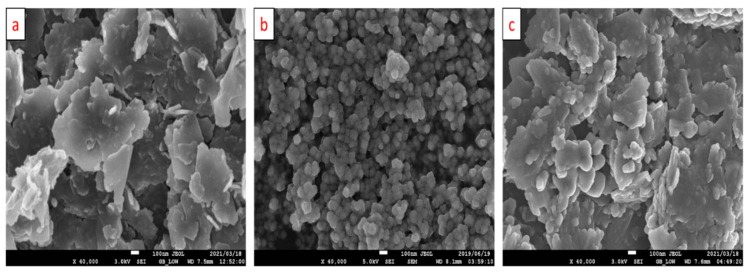
HR-SEM images of RGO nanosheets (**a**), MnO_2_ nanoparticles (**b**) and the MnO_2_@RGO nanocomposite (**c**).

The HR-SEM images in Figure 2 illustrates the change in morphology during the fabrication of the MnO_2_@RGO sensing electrode. The RGO surface (Figure 2a) shows the thermally exfoliated nanolayers with a thickness ranging between 15 and 25 nm and a lateral size ranging between 55 and 450 nm. The spherical MnO_2_ nanoparticles (Figure 2b) appear to be slightly agglomerated, but with a uniform shape. The simultaneous deposition of the MnO_2_ and RGO on the surface of the GCE yielded to the MnO_2_@RGO nanocomposite displayed in Figure 2c. Compared to the RGO, the MnO_2_@RGO nanocomposite shows nanolayers with some nanoparticles on their surface, signifying that the RGO nanosheets act as a matrix for connecting the MnO_2_ nanoparticles. Furthermore, the nanosheets in the MnO_2_@RGO nanocomposite appear to be thicker (21–36 nm) and larger in size (62–480 nm), due to the MnO_2_ nanoparticles amalgamated on the surface of the RGO.

The EDX elemental mapping and composition of RGO nanosheets (Figure S1) show the presence of C (84.04%) and O (15.96%) across the entire surface (Figure S1a–c), with no impurities on the EDX spectrum (Figure S1d). MnO_2_ nanoparticles (Figure S2) display the presence of Mn (27.61%) and O (24.76%) distributed evenly across the entire surface (Figure S2a–c, with C (47.38%) observed in the EDX spectrum from coating of the material, alongside traces of Al (0.14%) and Cl (0.11%) (from the sample stubs and remains from the initial salts after washing with deionized water, respectively) (Figure S2d). The MnO_2_@RGO nanocomposite (Figure S3) displays the presence of Mn (35.97%), O (16.88%), and C (46.60%) distributed across the surface (Figure S3a–d), with traces of Al (0.55%) from the sample stabs. In addition to showing the distribution of atoms on the surface of the materials, these results further confirm the successful synthesis of RGO nanosheets, MnO_2_ nanoparticles, and MnO_2_@RGO nanocomposite.

### 3.3. Transmission Electron Microscopy (TEM)

**Figure 3:**
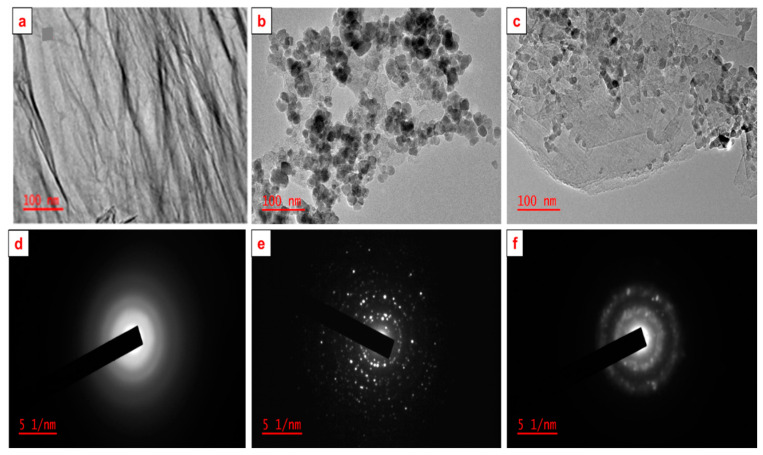
TEM images of RGO nanosheets (**a**), MnO_2_ nanoparticles (**b**) and the MnO_2_@RGO nanocomposite (**c**), with corresponding SAED patterns (**d**–**f**).

To gain further insights on the morphology of the RGO nanosheets, MnO_2_ nanoparticles and MnO_2_@RGO nanocomposite, TEM was explored (Figure 3). The RGO nanosheets (Figure 3a) display the typical wrinkled pattern. The uniformly spherically shaped MnO_2_ nanoparticles are displayed in Figure 3b. Interestingly, the uniformly shaped MnO_2_ nanoparticles adhere to the surface of the RGO nanosheets after their simultaneous deposition (Figure 3c). This then suggests that there were strong interactions between RGO sheets and MnO_2_ particles, which might then prevent the leaching of MnO_2_ nanoparticles in solution [3]. This observation corroborates with the HR-SEM results. Expectedly, three amorphous diffraction rings corresponding to the unique (002), (110), (211) facets on the XRD of the RGO were observed in the selected area electron diffraction (SAED) pattern (Figure 3d). Furthermore, multiple polyatomic rings with bright spots were observed on the SAED pattern of the MnO_2_ nanoparticles, with rings corresponding to the (110), (211), (301), and (521) facets on the XRD of the MnO_2_ nanoparticles. The bright spots are indicative of the intense crystallinity of MnO_2_ nanoparticles. Remarkably, the MnO_2_@RGO nanocomposite display the amorphous polyatomic rings with bright spots signifying the presence of both RGO nanosheets and MnO_2_ nanoparticles. Moreover, the diffraction rings observed correspond to the (002), (110), (301), and (521) facets on the XRD of MnO_2_@RGO nanocomposite, showing the character of both MnO_2_ nanoparticles and RGO nanosheets. This further confirms the successful integration of MnO_2_ nanoparticles on the surface of RGO nanosheets [42].

### 3.4. Fourier Transform Infrared Spectroscopy (FTIR)

**Figure 4:**
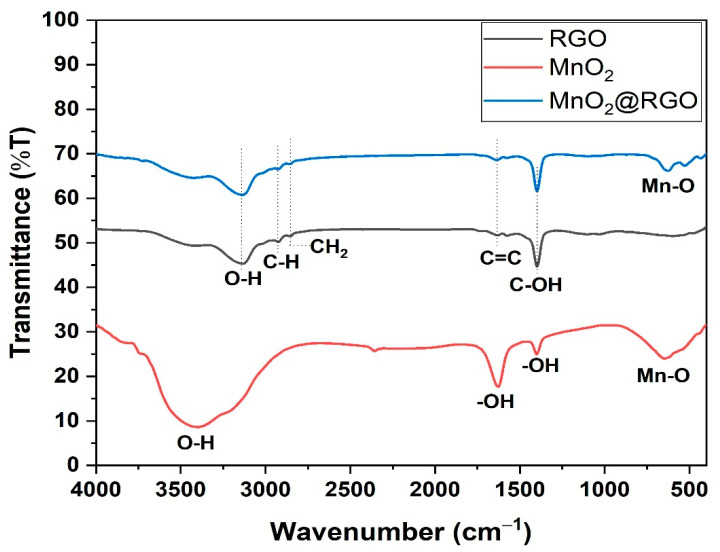
FTIR spectra of RGO nanosheets, MnO_2_ nanoparticles and MnO_2_@RGO nanocomposite.

The functional groups present in the synthesized RGO nanosheets, MnO_2_ nanoparticles, and MnO_2_@RGO nanocomposite were determined using the FTIR (Figure 4). The FTIR spectra of the MnO_2_ nanoparticles display a broad band around 3404 cm^−1^ corresponding to the -OH vibration mode of absorbed water [43]. The vibration bands at 1629 and 1398 cm^−1^ can be ascribed to the -OH stretching of the adsorbed water and the metallic hydroxide (Mn-OH) formed during the electrodeposition process, respectively [42,43,44]. The broad band around 647 cm^−1^ was attributed to the stretching Mn-O bond in MnO_2_ nanoparticles [39,43,44]. The RGO nanosheets display the broad -OH band around 3140 cm^−1^ with a negligible shift in position when compared to the -OH of the MnO_2_@RGO nanocomposite [42]. The bands at 2923 and 2862 cm^−1^ correspond to the -CH and CH_2_ groups in the RGO structure, respectively. Similarly, these groups showed a negligible change in position on the MnO_2_@RGO nanocomposite [38,39,43]. Likewise, the bands around 1634 and 1393 cm^−1^ correspond to the C=C and C-O on the backbone of RGO, with a similar position and negligible change also observed on the MnO_2_@RGO nanocomposite [33,34,42]. The presence of the Mn-O stretching vibration around 633 cm^−1^ on the nanocomposite suggests the successful integration of the MnO_2_ with the RGO nanosheets [42,43,44]. Although the deposition of the RGO nanosheets and MnO_2_ nanoparticles were done simultaneously to form the MnO_2_@RGO nanocomposite, the FTIR results obtained suggest that the MnO_2_@RGO nanocomposite chemical structure resembles that of the RGO nanosheets, with MnO_2_ embedded on the structure of the RGO nanosheets. These results agree with the HR-SEM results showing the morphology of RGO as more dominant on the MnO_2_@RGO nanocomposite, suggesting a more conductive material formed [3,42].

### 3.5. Nitrogen Absorption-Desorption Studies

The BET properties of RGO nanosheets, MnO_2_ nanoparticles, and MnO_2_@RGO nanocomposite were determined from the N_2_ adsorption-desorption isotherms measured at 77K (Table 1). MnO_2_@RGO nanocomposite displayed a specific surface area of 148.7 m^2^/g which was by far higher than the 77.5 and 54.1 m^2^/g specific surface area of MnO_2_ nanoparticles and RGO nanosheets, respectively. The high increase of the surface area can be attributed to the integration of MnO_2_ nanoparticles with RGO nanosheets. Expectedly, MnO_2_@RGO nanocomposite displayed higher average pore size and the total pore volume than both RGO nanosheets and MnO_2_ nanoparticles as summarized in Table 1.

### 3.6. Electrochemical Performance of the Sensing Materials

**Figure 5:**
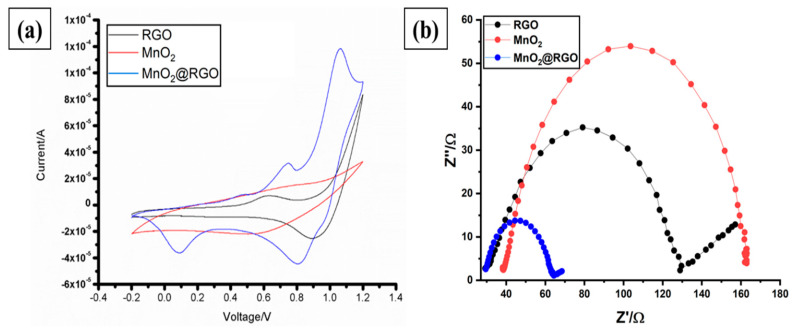
(**a**) CV voltammograms recorded at potential range of −0.2 to 1.2 V, at a scan rate of 25 mV s^−1^ in 0.1 M KCl (**b**) Nyquist EIS plots (**b**) obtained after the electrodeposition of RGO nanosheets, MnO_2_ nanoparticles and MnO_2_@RGO nanocomposite on the GCE surface.

The electrochemical performance of the functional MnO_2_@RGO nanocomposite was evaluated on a typical three-electrode setup. The GCE can be defined as the current collector, whereas the RGO nanosheets, MnO_2_ nanoparticles, and MnO_2_@RGO nanocomposite are the sensing materials. Prior to trace metal determinations, the bare GCE was immersed in the suspension of each sensing material and cyclic voltammetry (CV) was employed for the electrodeposition of the sensing materials on the polished GCE surface in a potential range of −0.2 to 1.2 V, at a scan rate of 25 mV s^−1^ in 0.1 M KCl. The corresponding cyclic voltammograms obtained from the electrodeposition of RGO nanosheets, MnO_2_ nanoparticles, and MnO_2_@RGO nanocomposite modified electrodes are displayed in Figure 5a. As seen, the MnO_2_@RGO nanocomposite modified GCE showed persistent enhancement currents when compared to the RGO nanosheets modified GCE and MnO_2_ nanoparticles modified GCE. This indicates that the incorporation of MnO_2_ nanoparticles on the RGO nanosheets improves the sensing element of the GCE. Evidently, the charge-transfer resistance of the prepared materials was studied, and the EIS measurements for the various electrodes are represented by the Nyquist plots in Figure 5b. Although all the materials display semicircles of different diameters within the high and low frequency regions, the Nyquist plot of the MnO_2_@RGO nanocomposite modified GCE exhibits a very small semicircle, indicating the lowest charge transfer resistance (Rct) (34.71 Ω) than the RGO nanosheets (99.54 Ω) and MnO_2_ nanoparticles (124.61 Ω). The low Rct of MnO_2_@RGO nanocomposite modified GCE might be ascribed to the high electrocatalytic activity of the incorporated MnO_2_ layer [3] and the increased porous structure of the composite.

Furthermore, the solution resistance (Rs) was determined as 29.39 Ω, 38.23 Ω, and 29.41 Ω for the RGO nanosheets, MnO_2_ nanoparticles, and MnO_2_@RGO nanocomposite, respectively. These results indicate a strong interaction between the electrode–electrolyte interface region of the MnO_2_@RGO nanocomposite GCE resulting from the highly porous surface of the MnO_2_@RGO nanocomposite, as confirmed by the BET results (Table 1). In addition to the semicircle observed, the MnO_2_@RGO nanocomposite modified GCE display the sharply reduced Warburg impedance (W) initially observed from the low frequencies of the Nyquist plot of the RGO nanosheets. Although MnO_2_ nanoparticles display the lowest conductivity, their integration with the conductive RGO nanosheets greatly improves the electron transport capacity and therefore increases the sensitivity, as shown by the increased current response observed from the cyclic voltammograms (Figure 5a). Following these results, the MnO_2_@RGO nanocomposite modified GCE was therefore employed to evaluate the response signals of the trace Cd(II), Zn(II), and Cu(II) in aqueous solutions.

**Figure 6:**
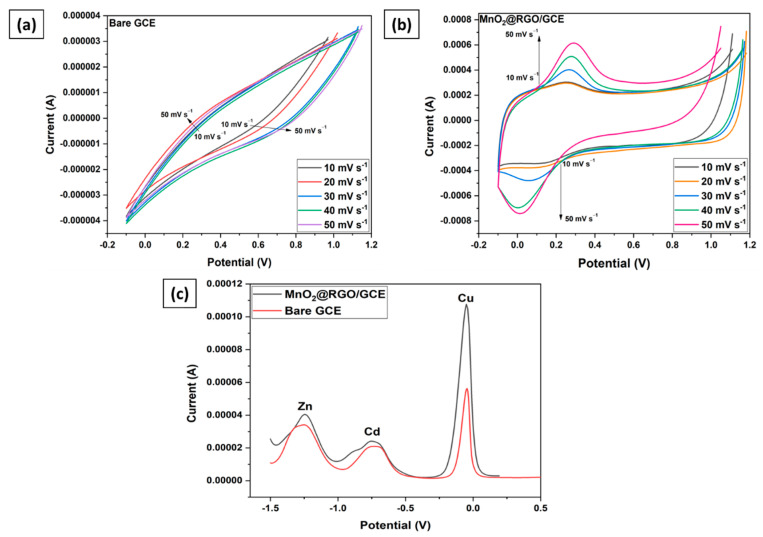
Cyclic voltammograms at different scan rates for (**a**) Bare GC electrode, (**b**) MnO_2_@RGO nanocomposite modified GCE; Differential pulse voltammograms for the detection of Cu(II), Cd(II), Zn(II) using bare GCE and MnO_2_@RGO nanocomposite modified GCE (**c**).

Prior to the determination of Cu(II), Cd(II), Zn(II) using DPASV, cyclic voltammograms were recorded at different scan rates (5–50 mV s^−1^) in an electrolyte of 0.1 M KCl, 5 mM [Fe(CN)6]^4−^, and 5 mM [Fe(CN)6]^3−^ to evaluate the effective electroactive surface area (Aeff) on the bare GCE (Figure 6a) and MnO_2_@RGO nanocomposite modified GCE (Figure 6b) [3]. The Aeff values were determined using the Randles–Sevcik equation (Equation (1)):

(1) $$I_{p}=2.69\times 10^{5}n^{3/2}A_{eff}D_{0}{}^{1/2}Cv^{1/2}$$

where: *n* = number of electrons, *A_eff_* = effective electroactive surface area (cm^2^), *D*_0_ = diffusion coefficient ((cm^2^/s) of [Fe(CN)6]^4−/3−^, *C* = concentration of the solution (mol/cm^3^), and *v* = scan rate (V/s). Using *n* = 2, *C* = 5 mM, *D*_0_ = 7.6 × 10^−6^ cm^2^ s^−1^, and the slope (Ip/v^1/2^), the Aeff values of bare GCE and MnO_2_@RGO nanocomposite modified GCE were calculated as 1.24 and 3.89 cm^2^, respectively. Notably, the Aeff values of the nanocomposite modified GCE is considerably larger than the bare GCE.

The differential pulse voltammograms showing the responses of the bare GCE and the MnO_2_@RGO nanocomposite modified GCE on the detection of Cu(II), Cd(II), and Zn(II) were presented in Figure 6c. It can be comprehended that the MnO_2_@RGO nanocomposite modified GCE produces a large stripping signals compared with the bare GCE. Furthermore, the influence of the oxygen interference was negligible with favorable signal-background characteristics. The superior electrocatalytic activity of the MnO_2_@RGO nanocomposite modified GCE achieved can be attributed to the synergistic coupling effect emancipating from the uniformly distributed small MnO_2_ nanoparticles on the RGO nanosheets surface [45]. Although DPASV resulted in satisfactory current responses on the detection of Cu(II), Cd(II), and Zn(II) in synthetic samples, the method was optimized to ensure the accurate determination of the trace metals in real water samples.

### 3.7. Optimization of the DPASV Experimental Parameters

**Figure 7:**
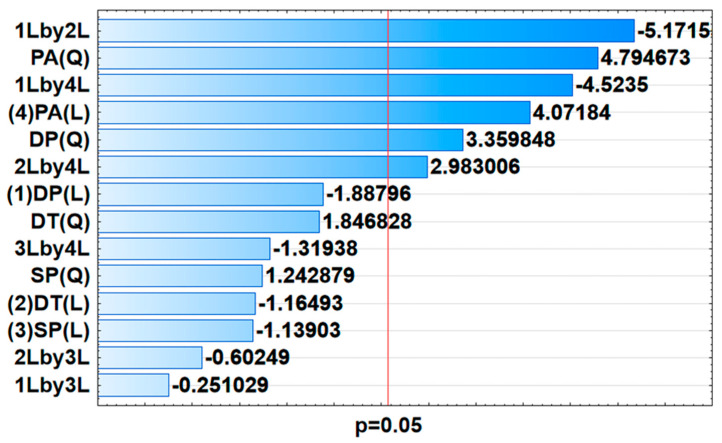
Pareto charts showing the significance of independent factors and their interactions for the determination of Zn(II).

The influential parameters (deposition time (DT), deposition potential (DP), pulse amplitude (PA), and step potential (SP)) affecting the determination of Cu(II), Cd(II), Zn(II) using MnO_2_@RGO nanocomposite modified GCE were optimized using the small-central composite design (SCCD). The design of the experiments (DOE) is shown in Table S1, alongside the current response of each element when subjected to the influential parameters (Table S2). Using the results obtained in Table S2, the significance of each factor was examined using analysis of variance (ANOVA). The ANOVA results were represented in the form of pareto charts (Figure 7, Figures S4 and S5). Considering the fact that ANOVA is a linear model, only linear factors (factors with the index L) were considered, while quadratic factors (factors with the index Q) were not considered on the pareto chart analysis [46]. Evidently, it can be clearly seen in Figure 7 that the three interactions (1. DT and DP; 2. DP and PA; 3. DT and PA), alongside the independent PA factor were significant at 95% confidence level for Zn. However, Cd (Figure S4) only shows one interaction between DP and DT as significant at 95% confidence level, alongside PA, which is the only independent factor that was significant. The pareto chart for Cu (Figure S5) shows that none of the factors were significant at 95% confidence level. However, PA and SP were found to be important and influential on analytical response. It can therefore be clearly seen that the PA has the overall most significant effect on the analytical response (peak current).

**Figure 8:**
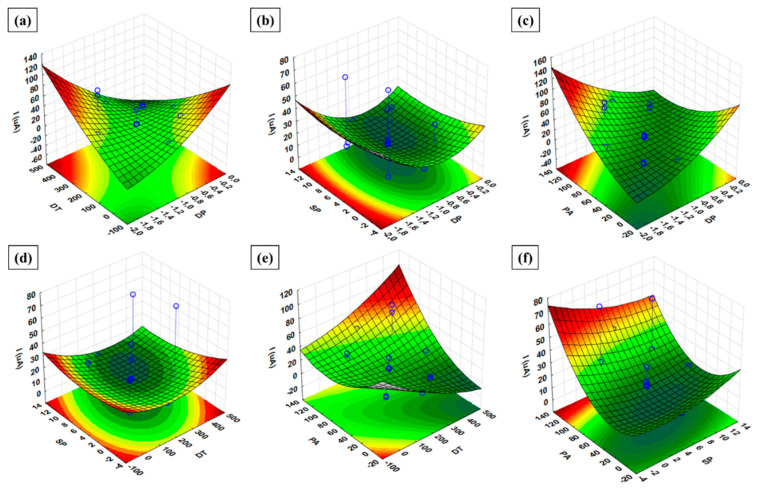
RSM plots for the optimization of Zn(II).

The response surface methodology (RSM) was used to investigate the interaction and the quadratic effects of the main parameters DP, DT, and SP and PA, using the data generated from the experimental SCCD, resulting in 3D surface plots for the Zn(II) optimization (Figure 8). Clearly, from Figure 8a, increasing DT to any value greater than 250 results in higher current response, even at low DT values. Similarly, the DP at higher values (0 to −0.7) lead to higher current response at low DT values, signifying the significance of the interaction of DP and DT. Furthermore, the interaction of SP with DP in Figure 8b clearly shows that any given SP value (from −4 to 1.4/higher) interacting with low DP values (from −1.6 to −2.0/lower) result to higher current responses. Figure 8c shows that increasing the PA to higher values (>90) leads to a higher current response at lower or higher DP values, indicating the significance of PA, alongside the insignificance of DP. Figure 8d shows that a higher current response can be achieved at any SP value (−4 to 14/higher) interaction with higher DT (>350) or lower DT (<0), indicating the significance of the interaction of SP and DT at given values. Furthermore, low PA and low DT (both below 0) give high response as shown in Figure 8e. Finally, the interaction of PA and SP shows that higher PA (>110) correspond to higher response at any given SP, indicating the significance of PA and the insignificance of SP at given values (Figure 8f). The results obtained in these RSM plots, noticing the significant interactions, correspond to the pareto chart analysis.

The RSM plots for Cd(II) are shown in Figure S6. In Figure S6a, it can be seen that at higher DT values and higher DP values, there is a higher current response. Figure S6b shows that a DP between −1.6 to −0.4 and any SP between the given DP range leads to a higher current response. Furthermore, higher DP (>−1.8) and a higher PA (>120) interaction leads to a higher current response as shown in Figure S6c. DT between 0 and 350 interacting with any SP between the DP values given result to a higher current response (Figure S6d). Any DT value interacting with PA (<0) gives a maximum response (Figure S6e). Lastly, any SP value interacting with PA (<0) gives a maximum response (Figure S6f). These results are in agreement with the pareto chart analysis, as SP and DT were clearly insignificant. The RSM plots of Cu(II) shown in Figure S7 show a similar pattern with the RSM plots of Cd(II). Subsequently, DP, DT and SP were clearly insignificant on the current response, thus agreeing with the pareto chart analysis.

**Figure 9:**
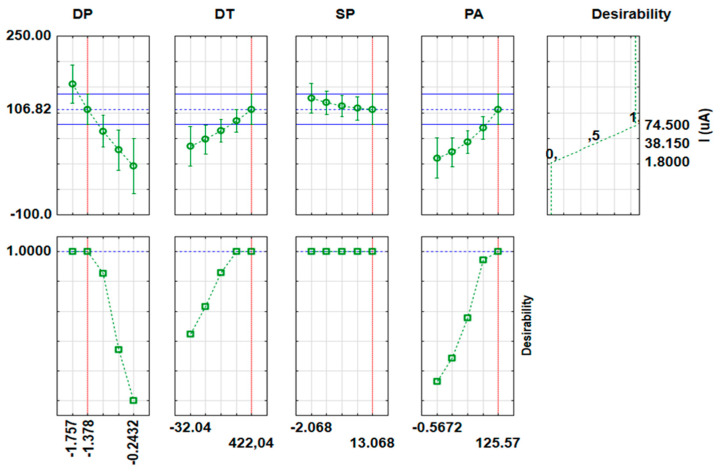
Profiles for predicted values and desirability of Zn(II).

The simultaneous estimations of the optimum conditions leading to the maximum current response were evaluated using the desirability function (DF) for all the parameters and metals (Figure 9, Figures S8 and S9). The desirability function takes values in the range 0–1, with 0 indicating a 1.8 µA current response; 0.5 indicating a 38.15 µA current response; and 1 showing a 74.50 µA current response for the Zn optimization (Figure 9). Similarly, on the Cd optimization, 0 indicates a 0.30 µA current response; 0.5 indicates a 17.85 µA current response; and 1 shows a 35.40 µA current response. Likewise, on the Cu optimization, 0 indicates a 3.00 µA current response; 0.5 indicates a 68.70 µA current response; and 1 shows a 134.40 µA current response. Evidently, from the desirability profiles, SCCD and RSM results, it can be concluded that the optimum conditions yielding the greatest current response for simultaneous determination of Cd(II), Cu(II), and Zn(II) were −1.5 V, 330 s, 25 mV, and 10 mV for DP, DT, PA, and SP, respectively.

### 3.8. Analytical Figures of Merit

Under optimum conditions, calibration curves from the differential pulse voltammograms using MnO_2_@RGO/GCE were studied for different concentrations of each metal ion (Cu, Zn and Cd). The limits of detection (LOD = 3Sb/m) and the limits of quantification (LOQ = 10Sd/m) are presented in Table 2, where Sd and m are standard deviation of the blank signal (n = 20) and the slope of the calibration curve, respectively. The intraday (repeatability) precision of the MnO_2_@RGO/GCE was investigated by performing 15 measurements of the standard solution of Cd, Cu and Zn ions at a concentration of 25 μg L^−1^. Additionally, the interday (reproducibility) precision was evaluated by preparing five MnO_2_@RGO/GCE from the same batch in five consecutive working days. The MnO_2_@RGO/GCE were used for the determination of 25 μg L^−1^ of metal ions. The precisions were expressed in terms of relative standard deviation (RSD) and the results are summarized in Table 2. Consequently, the intraday and interday precisions for MnO_2_@RGO/GCE were acceptable, as they were less than 5%. Moreover, the MnO_2_@RGO/GCE was found to be reusable for four weeks and after the response of the MnO_2_@RGO/GCE decreased.

Subsequently, the comparison of MnO_2_@RGO/GCE with other reported modified electrodes in the determination of Cd, Cu and Zn ions is presented in Table 3. It can be seen from the table that the LOD and sensitivity towards determination of Zn improved. Additionally, it can be perceived that the present method exhibited a wider dynamic range and better or similar precision compared those reported in the literature.

### 3.9. Surface Water (SW) Sample Analysis

The applicability and validity of the proposed MnO_2_@RGO/GCE was investigated by the application on the determination of Zn^2+^, Cu^2+,^ and Cd^2+^ ions in surface water samples. Initially, the accuracy of the developed method was assessed by analyzing three water samples spiked with 2.00, 5.00, and 10.0 μg L^−1^ concentrations of Cu(II), Zn(II), and Cd(II). The concentration of the target elements was achieved by comparing the values obtained by using the calibration curve method. The results and percentage recovery values are shown in Table 4. According to the results obtained, a good agreement between the added and an obtained analyte concentration was achieved. The percentage recoveries ranged from 97–99.9%. These results reveal that that the developed electrochemical sensor method has a relatively good accuracy in real water sample matrix.

The applicability of the developed electrochemical sensor for the simultaneous determination of Cd, Cu, and Zn in real water samples was investigated. The analytical results obtained are presented in Table 4 and Table 5. It can be seen in Table 5 that the results obtained by the developed electrochemical method show a good agreement with those obtained by the ICP-OES analysis. Therefore, these findings confirmed the applicability of the proposed method for precise and accurate simultaneous determination of Cd, Cu and Zn in real environmental water samples. In addition, these results revealed that the current electrochemical sensor has capabilities to determine the trace metal ions the real water samples without significant interferences.

### 3.10. Stability of the Electrochemical Sensor

**Figure 10:**
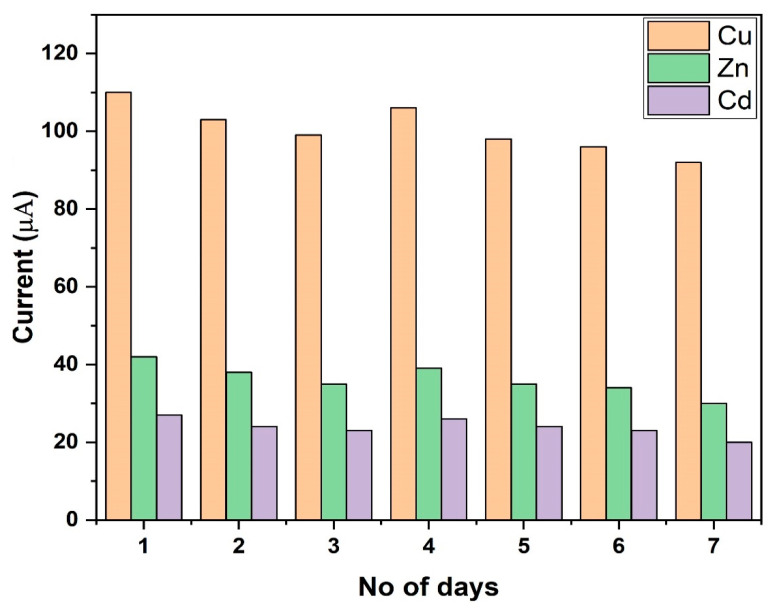
Long term stability of MnO_2_@RGO/GCE.

The stability of the modified GCE was investigated by repetitively measuring 25 μg L^−1^ of Cd, Cu, and Zn using the same modified electrode. These experiments were conducted under optimal experimental conditions. After every use, the MnO_2_@RGO nanocomposite modified GCE was immersed in 2 M nitric acid to dissolve any adsorbed metal ions and stored in the refrigerator at 4 °C. The experimental results for the long-term stability of the MnO_2_@RGO nanocomposite modified GCE are displayed in Figure 10. Evidently, the developed sensor was found to be stable as it retained about 95% of the initial current response in the determination of Cu(II), Cd(II), and Zn(II). This implies that the sensor could be used continuously for at least 7 days without any significant loss in performance.

## 4. Conclusions

This work reported on the determination of Cu(II), Cd(II), and Zn(II) using the fabricated MnO_2_@RGO/GCE modified electrode using DPASV. The electrodeposited MnO_2_@RGO/GCE exhibited high electrocatalytic activity owing to its increased surface area, low charge-transfer resistance at the electrolyte-electrode interface, and high selectivity to Cu(II), Cd(II), and Zn(II). Experimental parameters such as deposition potential, deposition time, pulse amplitude, and step potential were optimized using the small central composite design and the response surface methodology for Cu(II), Cd(II), and Zn(II). The experimental electrochemical results using the optimum conditions suggest that the MnO_2_@RGO nanocomposite enhances the current response on the detection of Cu(II), Cd(II), and Zn(II) in surface water. Additionally, the MnO_2_@RGO modified GCE exhibited several advantages, such as the simple preparation, reproducibility, and good stability. Furthermore, the fabricated electrochemical sensor proved to be a better alternative method that can be used for the determination of heavy metals in environmental matrices without the sample preparation step and can be used in routine analysis.

## Figures and Tables

**Table 1:** BET properties of RGO nanosheets, MnO_2_ nanoparticles, and MnO_2_@RGO nanocomposite.

Surface Properties	RGO Nanosheets	MnO_2_ Nanoparticles	MnO_2_@RGO Nanocomposite
BET surface area (m^2^/g)	54.1	77.5	148.7
Average pore size (nm)	14.1	18.8	26.6
Total pore volume (cm^3^/g)	0.19	0.33	0.77

**Table 2:** Analytical parameter of the MnO_2_@RGO/GCE for the determination of Cd, Cu, and Zn ions.

Analytical Parameters	Cd	Cu	Zn
DLR (μg L^−1^)	LOQ-700	LOQ-600	LOQ-600
R2	0.9967	0.9990	0.9987
RLE	$I(\mu A)=0.2089C_{Cd}(\mu g\;L^{-1})-2.929$	$I(\mu A)=0.5639C_{Cu}(\mu g\;L^{-1})+25.35$	$I(\mu A)=0.2465C_{Zn}(\mu g\;L^{-1})-1.755$
LOD (ng L^−1^)	15.1	9.3	13.7
LOQ (ng L^−1^)	50.3	31.0	44.3
Intraday (%RSD)	2.1	1.5	2.5
Interday (%RSD)	3.9	4.0	4.5

**Table 3:** Comparison of analytical parameters of the different modified electrodes reported for the determination of Cd, Cu, and Zn ions.

Electrode/Method	Analytes	LOD (μg L^−1^)	LOQ(μg L^−1^)	DLR (μg L^−1^)	RSD %	Ref
Mo_6_S_x_I_9-x_NWs/GCE/DPASV	Cu and Cd	0.015		0.50–240	3.3–5.1	[27]
BiFE/GCE	Cu	0.028		5–110	-	[9]
Nafion/Bi/NMC/GCE/DPASV	Cu, Cd, Zn	0.93–1.07		2–100	7.5	[47]
RGO-CS/PLL/GCE	Cd and Cu	0.01–0.02		0.05–10.	4.4	[25]
Nano-Al_2_O/GCE/DPV	Zn	0.026		0.08–150	3.39	[48]
CS/AuNPs/GR/GCE	Cd	0.002		0.1–0.9	2.4	[49]
CB–Nafion–GCE	Cd	0.9		0.6–112	3.13	[50]
MnO_2_@RGO/GCE/DPV	Cd, Zn and Cu	0.002–0.015	0.021–0.050	0.05–700	1.5–4.5	This study

**Table 4:** Results for Cd, Cu and Zn ions determination in spiked surface water samples obtained under the optimum conditions (N = 5).

Samples	Added	Cd		Cu		Zn	
		Found	%R	Found	%R	Found	%R
SW1	0	1.32 ± 0.05	-	56.7 ± 0.9	-	126.5 ± 1.8	-
	2.0	3.27 ± 0.02	97.5	58.7 ± 1.0	99.0	128.5 ± 1.3	98.5
	5.0	6.18 ± 0.12	97.2	61.6 ± 0.8	99.0	131.4 ± 1.6	98.8
	10.0	11.2 ± 0.17	98.5	66.6 ± 0.9	99.0	±1.5	99.8
SW2	0	2.45 ± 0.02	-	102.4 ± 1.5	-	337.1 ± 3.1	-
	2.0	4.43 ± 0.07	99.0	104.4 ± 1.4	99.0	339.0 ± 2.3	97.0
	5.0	7.37 ± 0.06	98.2	107.3 ± 1.6	99.2	342.1 ± 3.1	99.2
	10.0	12.7 ± 0.80	99.1	112.4 ± 1.2	99.6	347.0 ± 2.5	99.3
SW3	0	1.66 ± 0.06	-	125.9 ± 1.3	-	231.0 ± 2.4	-
	2.0	3.63 ± 0.05	98.5	127.9 ± 1.1	98.5	232.0 ± 2.5	99.0
	5.0	6.61 ± 0.16	99.0	130.9 ± 1.3	99.6	235.0 ± 2.6	99.4
	10.0	11.6 ± 0.23	99.4	135.8 ± 1.5	99.0	240.9 ± 2.7	99.4

**Table 5:** Simultaneous determination Cd, Cu, and Zn ions in real surface water samples using MnO_2_@RGO/GCE/DPASV method (N = 5).

Samples	Concentration (µg L^−1^)			ICP-OES (Concentration (µg L^−1^)		
	Cd	Cu	Zn	Cd	Cu	Zn
SW4	<LOD	130.4 ± 1.2	231.6 ± 3.0	<LOD	129.5 ± 2.3	231.6 ± 3.0
SW5	0.98 ± 0.01	75.4 ± 0.9	144.3 ± 1.8	1.00 ± 0.01	74.6 ± 2.2	143.5 ± 2.3
SW6	1.23 ± 0.06	29.6 ± 0.5	89.6 ± 0.8	1.21 ± 0.07	30.1 ± 1.3	90.3 ± 1.2
SW7	1.03 ± 0.02	238.1 ± 2.6	92.3 ± 1.0	1.00 ± 0.12	238.5 ± 1.6	91.8 ± 1.8
SW8	2.31 ± 0.05	77.5 ± 1.2	136.7 ± 2.1	2.34 ± 0.09	78.1 ± 1.8	137.0 ± 3.2

## Data Availability

Not applicable.

## Supplementary Materials

The following are available online at https://www.mdpi.com/article/10.3390/membranes11070517/s1, Figure S1: EDX mapping (a–c) with corresponding EDX elemental composition (d) of RGO nanosheets, Figure S2: EDX mapping (a–c) with corresponding EDX elemental composition (d) of MnO_2_ nanoparticles, Figure S3: EDX mapping (a–d) with corresponding EDX elemental composition (e) of MnO_2_@RGO nanocomposites, Figure S4: Pareto charts showing the significance of independent factors and their interactions for the determination of Cd(II), Figure S5: Pareto charts showing the significance of independent factors and their interactions for the determination of Cu(II), Figure S6: RSM plots for the optimization of Cd(II), Figure S7: RSM plots for the optimization of Cu(II), Figure S8: Profiles for predicted values and desirability of Cd(II), Figure S9: Profiles for predicted values and desirability of Cu(II) Table S1 Design of experiments for the small-central composite design (SCCD), Table S2 Central composite design current responses for Zn (II), Cd (II) and Cu (II).
