# Supplementary material for: MnO2@Reduced Graphene Oxide Nanocomposite-Based Electrochemical Sensor for the Simultaneous Determination of Trace Cd(II), Zn(II) and Cu(II) in Water Samples
Source: Membranes (Basel). 2021 Jul 9;11(7):517. doi: 10.3390/membranes11070517 (PMC8307232; doi:10.3390/membranes11070517)
Supplement: Supplementary file 1 [file membranes-11-00517-s001.zip › membranes-1288277-supplementary.pdf]

Type of the Paper (Supplementary data)

MnO_2@Reduced Graphene Oxide nanocomposite-based electrochemical sensor for the simultaneous determination of trace Cd(II), Zn(II) and Cu(II) in water samples

Tshimangadzo S. Munonde[1,2*], Siyamthanda H. Mnyipika[1], Philiswa N. Nomngongo[1,2,3*]

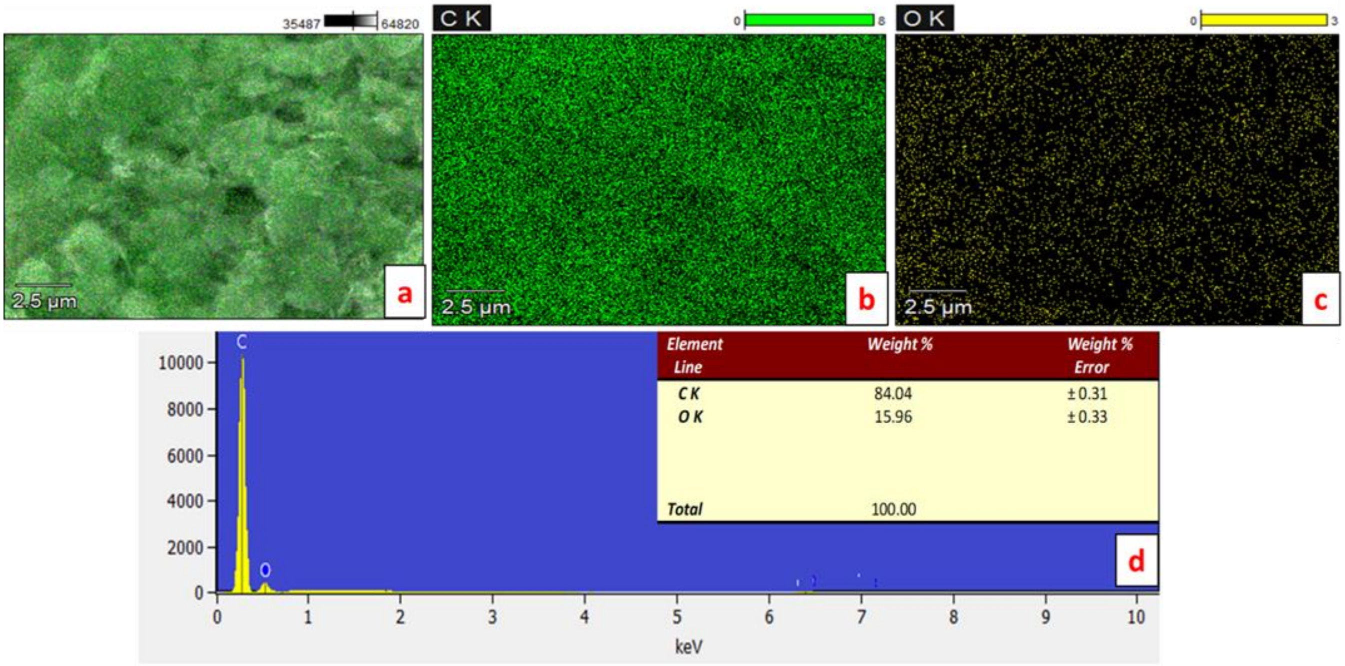

Figure S1 EDX mapping (a-c) with corresponding EDX elemental composition (d) of RGO nanosheets

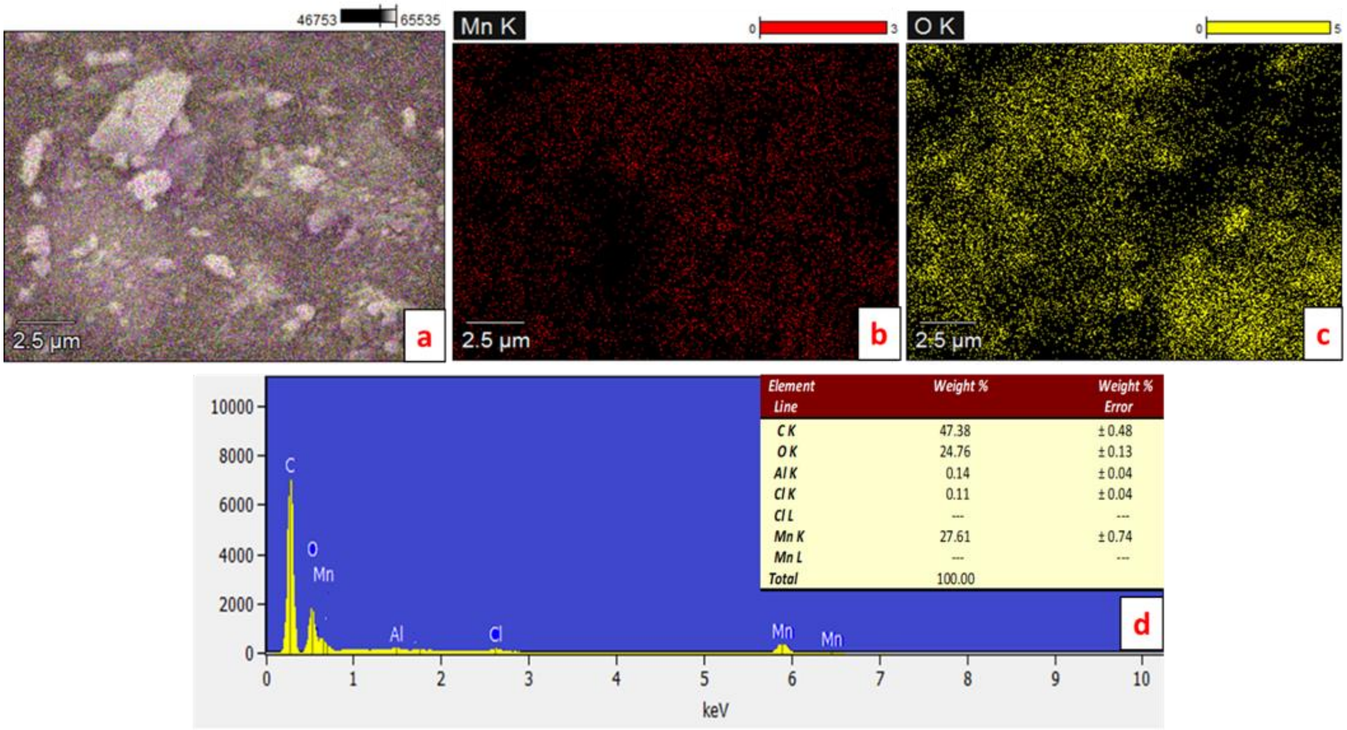

Figure S2 EDX mapping (a-c) with corresponding EDX elemental composition (d) of MnO_2 nanoparticles

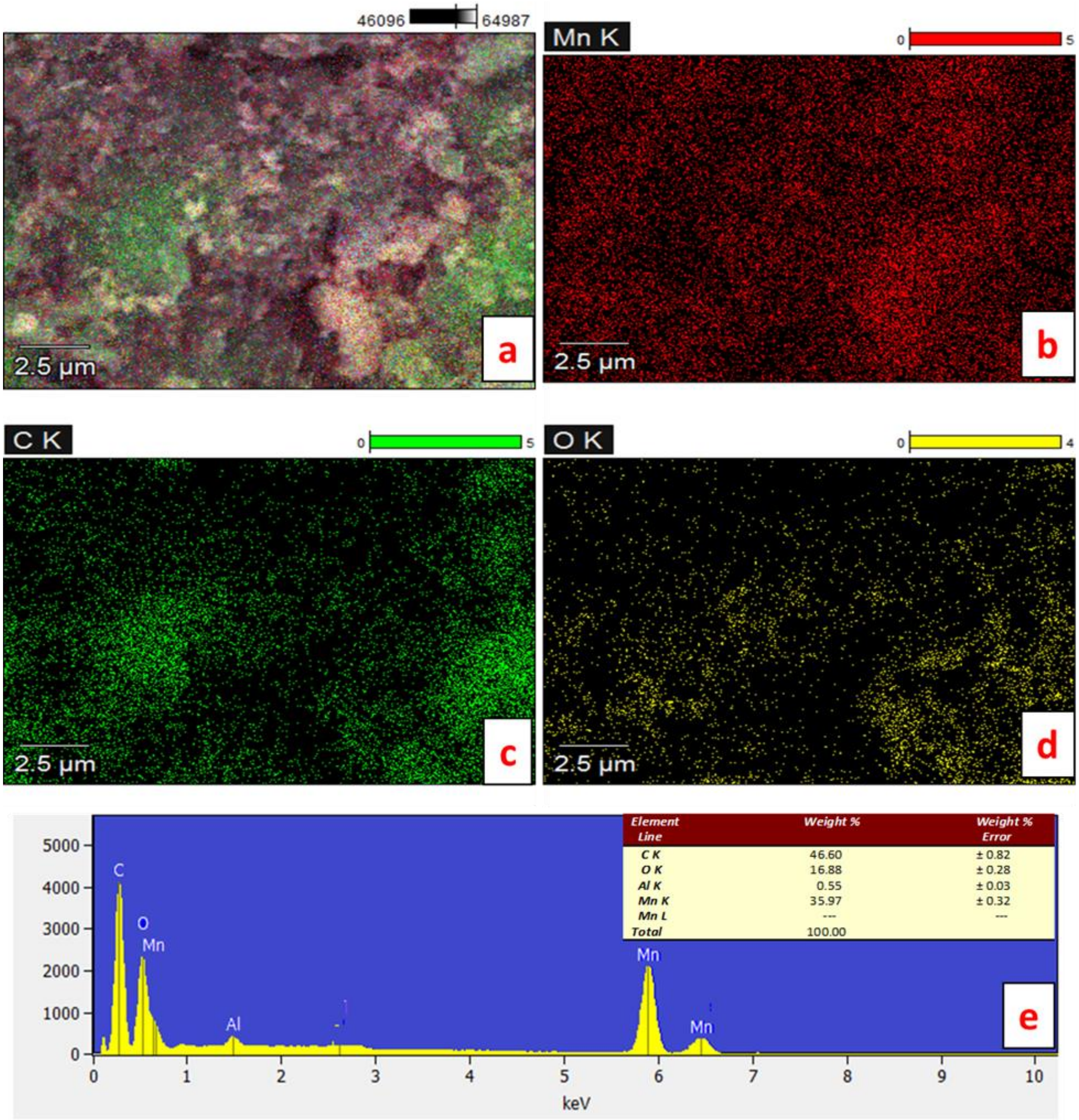

Figure S3 EDX mapping (a-d) with corresponding EDX elemental composition (e) of MnO_2@RGO nanocomposites

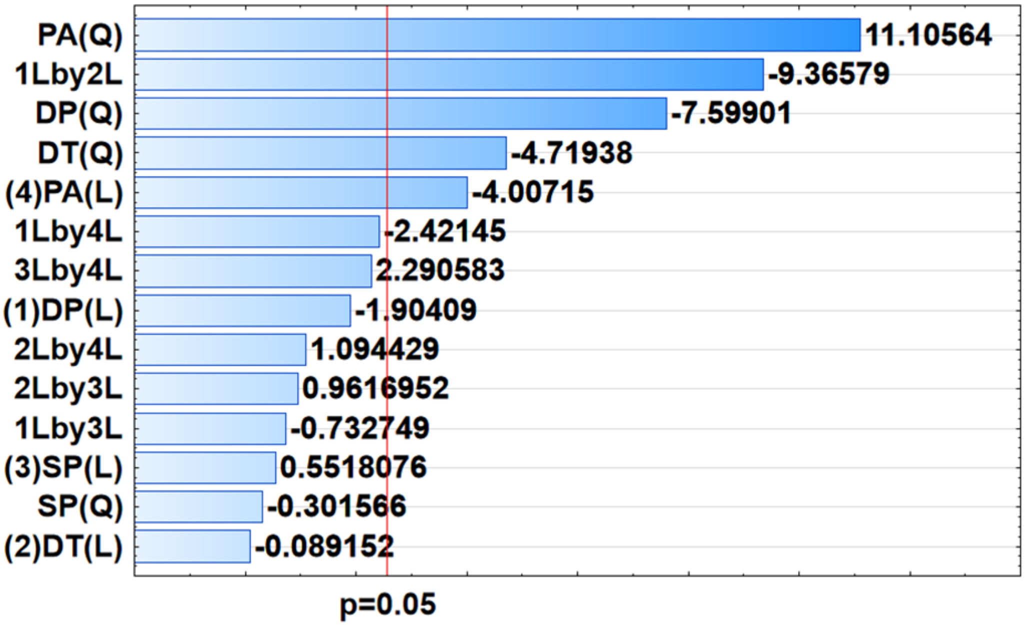

Figure S4 Pareto charts showing the significance of independent factors and their interactions for the determination of Cd(II)

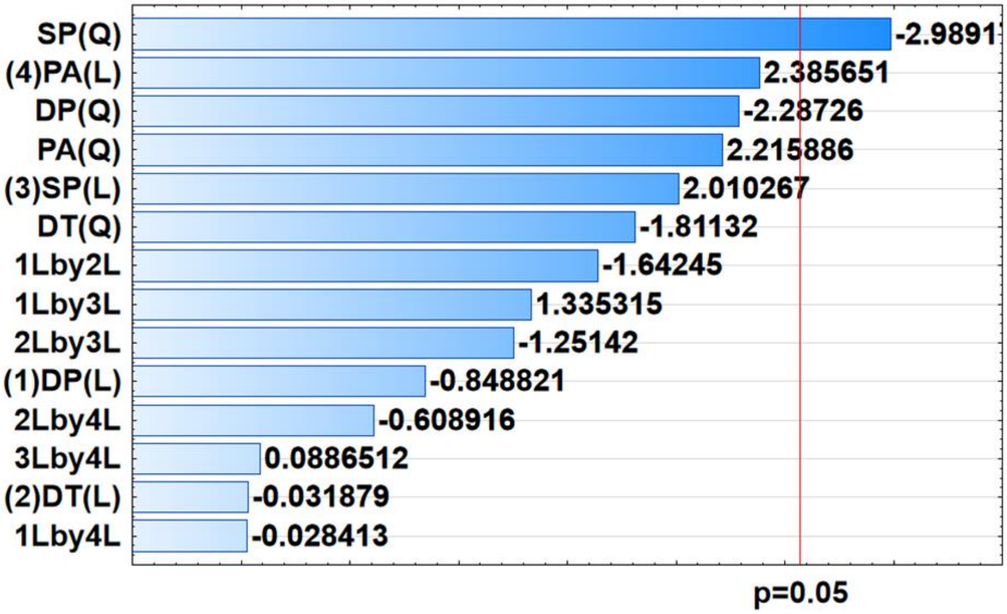

Figure S5 Pareto charts showing the significance of independent factors and their interactions for the determination of Cu(II)

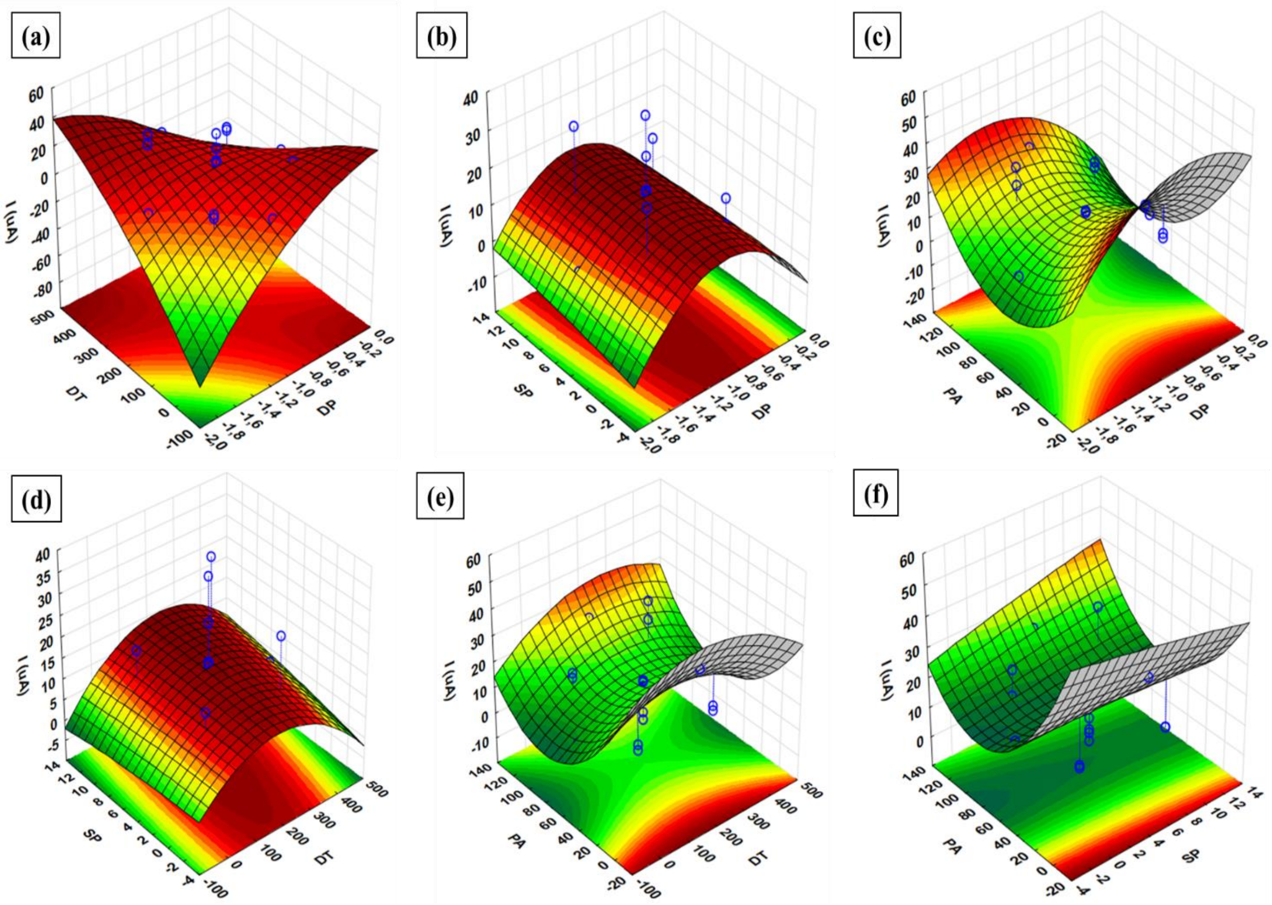

Figure S6 RSM plots for the optimization of Cd(II)

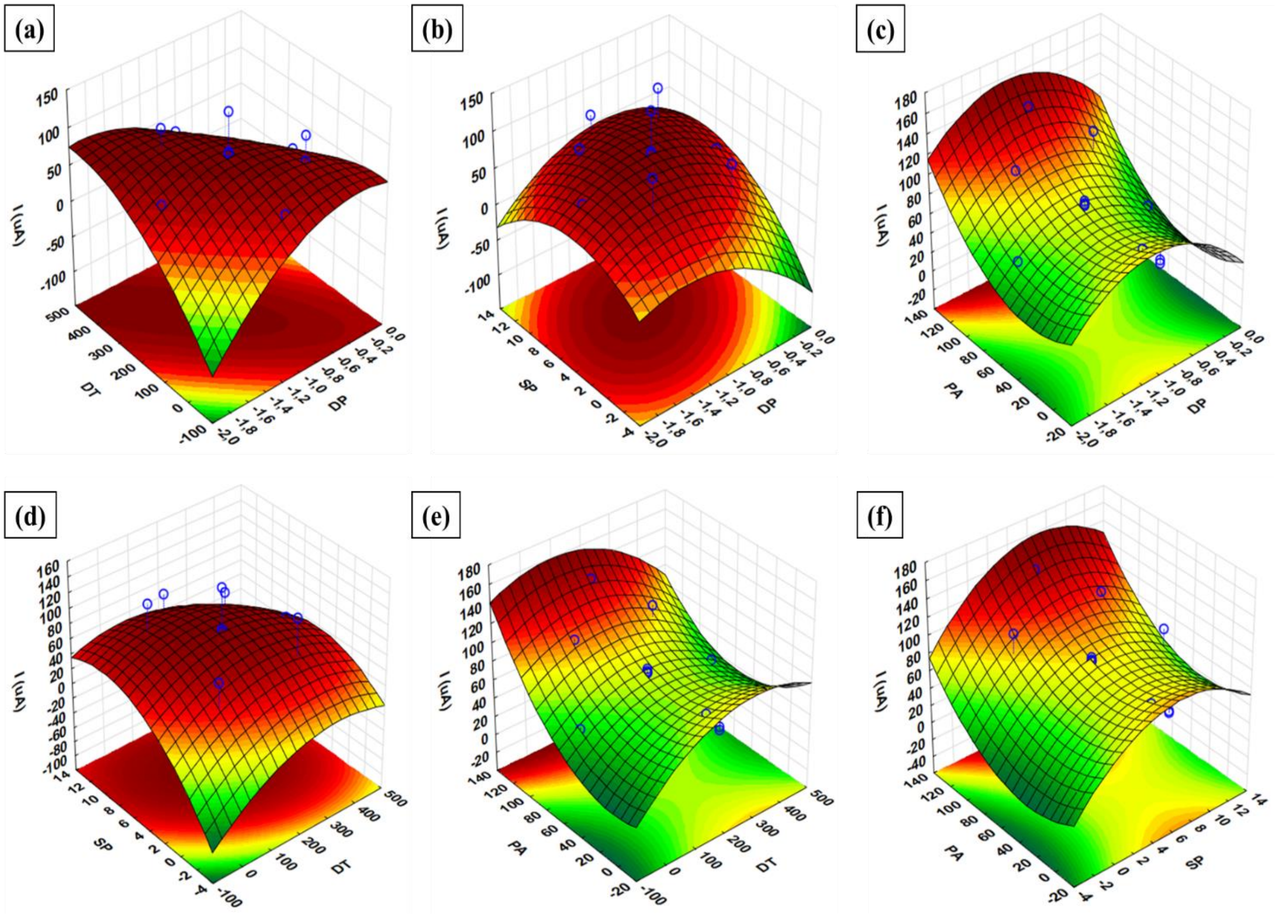

Figure S7 RSM plots for the optimization of Cu(II)

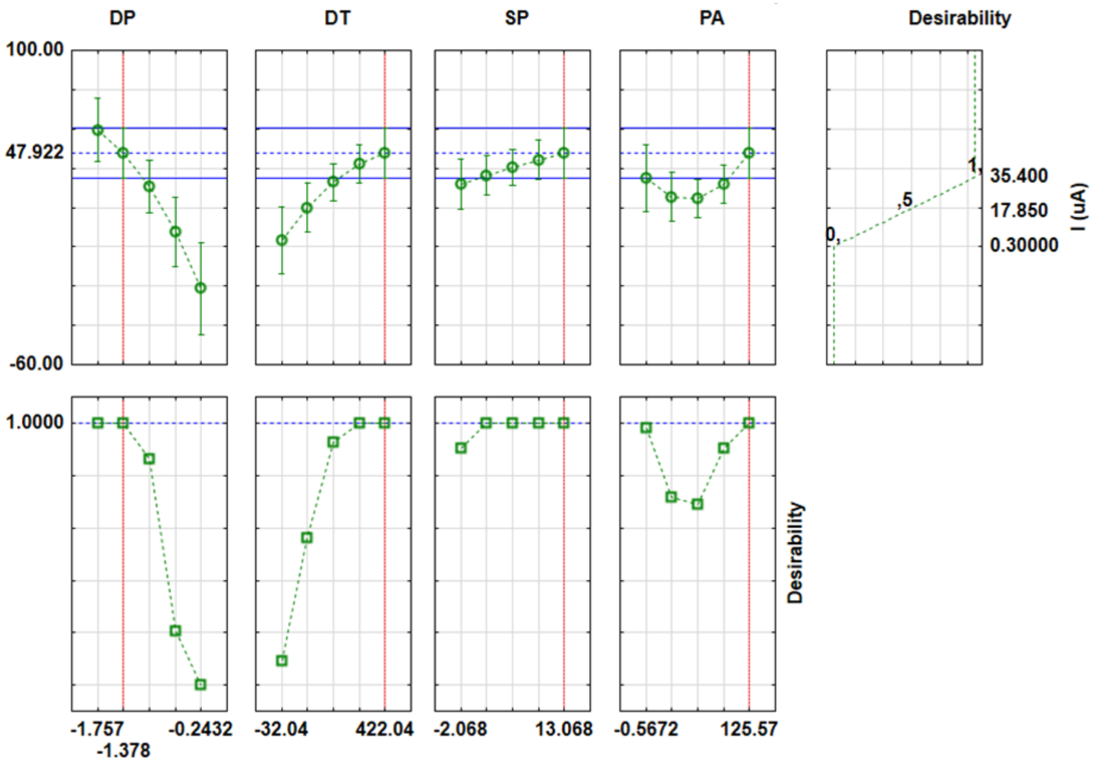

Figure S8 Profiles for predicted values and desirability of Cd(II)

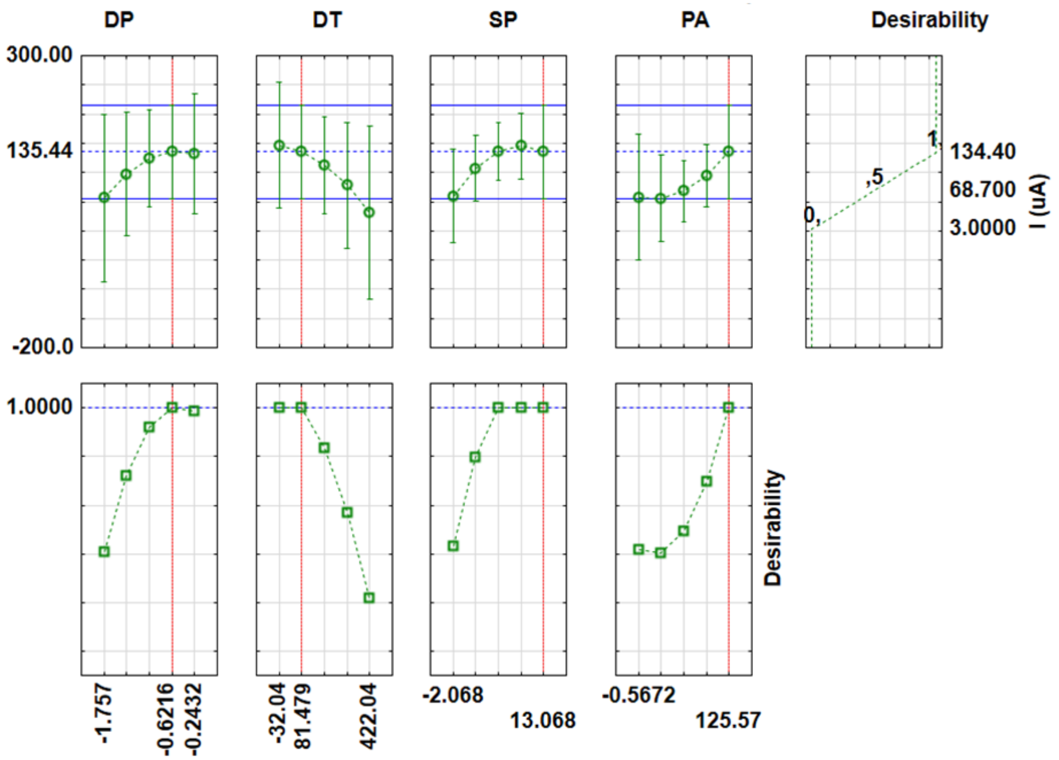

Figure S9 Profiles for predicted values and desirability of Cu(II)

Table S1 Design of experiments for the small-central composite design (SCCD)

Variable	Minimum	Central	Maximum
**DP**	-0.5	-1.0	-1.4
**DT**	60	195	330
**SP**	1	5.5	10
**PA**	25	62.5	100

Table S2 Central composite design current responses for Zn (II), Cd (II) and Cu (II)

EXPT	DP	DT	SP	PA	Zn (I, µA)	Cd (I, µA)	Cu (I, µA)
**1**	-0.5	330	10	25	1.8	5.9	22.1
**2**	-0.5	330	1	25	5.1	7.6	18.9
**3**	-0.5	60	10	100	23.7	16.7	105.0
**4**	-1.4	330	1	100	74.5	23.1	103.4
**5**	-0.5	60	1	100	31.7	14.6	69.3
**6**	-1.4	60	10	25	8.1	6.0	19.9
**7**	-1.4	330	10	100	63.7	30.2	72.6
**8**	-1.4	60	1	25	6.3	8.2	22.5
**9**	-1.7	195	5.5	62.5	29.1	3.1	53.4
**10**	-0.2	195	5.5	62.5	12.7	0.3	40.4
**11**	-1.0	-32	5.5	62.5	17.1	4.2	50.4
**12**	-1.0	422	5.5	62.5	14.3	7.9	55.5
**13**	-1.0	195	-2.1	62.5	13.3	13.0	3.0
**14**	-1.0	195	13.1	62.5	13.9	12.5	72.6
**15**	-1.0	195	5.5	-0.6	10.1	35.4	75.1
**16**	-1.0	195	5.5	125.6	41.8	24.7	134.3
**17 (C)**	-1.0	195	5.5	62.5	13.8	13.3	75.3
**18 (C)**	-1.0	195	5.5	62.5	13.2	14.9	77.2
**19 (C)**	-1.0	195	5.5	62.5	13.2	15.6	79.7
**20 (C)**	-1.0	195	5.5	62.5	12.7	15.6	75.2
